# Evaluation of Inflammatory and Oxidative Markers and Their Diagnostic Value in Schizophrenia

**DOI:** 10.3390/brainsci15111137

**Published:** 2025-10-23

**Authors:** Mehmet Güneş, Betül Uyar, Süleyman Dönmezdil, İbrahim Kaplan

**Affiliations:** 1 Department of Psychiatry, Faculty of Medicine, Dicle University, 21280 Diyarbakir, Turkey; m63gunes@gmail.com; 2 Department of Psychology, Faculty of Letters, Artuklu University, 47100 Mardin, Turkey; donmezdil@hotmail.com; 3 Department of Biochemistry, Faculty of Medicine, Dicle University, 21280 Diyarbakir, Turkey; dribrahimkaplan@hotmail.com

**Keywords:** schizophrenia, myleoperoxidase, catalase, oxidative stress, ınflammation

## Abstract

**Objective:** Schizophrenia is a chronic psychiatric disorder associated with increased oxidative stress. We aimed to investigate serum myeloperoxidase (MPO), catalase (CAT), and malondialdehyde (MDA) levels and their diagnostic value in schizophrenia. **Methods:** Sixty patients with schizophrenia, diagnosed according to DSM-V criteria, and 65 age- and sex-matched healthy controls were enrolled. Clinical severity was assessed with the Positive and Negative Syndrome Scale (PANSS) and Clinical Global Impression (CGI). Serum MPO and CAT were measured using ELISA, and MDA levels were determined spectrophotometrically. Receiver operating characteristic (ROC) analysis was performed to assess diagnostic performance. **Results:** Compared with controls, schizophrenia patients demonstrated significantly higher serum MDA (5.64 vs. 3.42 pg/mL, *p* < 0.001), MPO (77.25 vs. 31.42 ng/mL, *p* < 0.001), and CAT (22.06 vs. 6.58 ng/mL, *p* < 0.001) levels. Subgroup analysis revealed consistently increased values across patients receiving typical, atypical, or combined antipsychotics. ROC analysis indicated good diagnostic accuracy: AUC = 0.884 for MDA (cut-off: 3.79 pg/mL), AUC = 0.882 for MPO (cut-off: 34.56 ng/mL), and AUC = 0.875 for CAT (cut-off: 9.38 ng/mL), all *p* < 0.001. Combined analysis of MPO, CAT, and MDA yielded superior diagnostic performance (AUC = 0.995; sensitivity = 98.3%). MPO was positively correlated with PANSS-N scores (r = 0.275, *p* = 0.033), and both MPO and CAT were correlated with CGI severity scores. **Conclusions:** Elevated MPO, CAT, and MDA levels indicate increased oxidative stress in schizophrenia. MPO may also be associated with negative symptom severity. These findings suggest potential utility of oxidative stress biomarkers as adjunctive diagnostic tools, although results should be considered preliminary and validated in larger, drug-naïve, and longitudinal samples.

## 1. Introduction

Schizophrenia is a deteriorating disorder, affects approximately 1% of the general population and creates a high burden on an individual’s life by shortening lifespan and decreasing functionality [1,2]. Because of this burden, revealing the etiology and neurobiology of schizophrenia has been a major topic of interest in psychiatric research. At this point, being easily accessible and investigable makes circulating blood a valuable tissue in terms of biological marker detection. Several parameters like oxidative stress markers, antioxidant enzyme activities, inflammatory cytokines and microRNAs have been topics of interest in this context [3,4,5,6]. As aerobic organisms, the human body consumes high amounts of oxygen to survive. However, oxygen consumption results in the formation of oxidants such as reactive oxygen species, whereas antioxidant enzymes exist to neutralize and prevent harmful oxidative damage. Oxidative stress defines the imbalance between oxidants and antioxidant mechanisms [7]. The brain contains autoxidizable neurotransmitters such as norepinephrine and serotonin, has lipid-rich neuronal membranes, excitotoxic neurotransmitters like glutamate, and consumes high amounts of oxygen. With all these factors, the brain is vulnerable to oxidative stress caused by reactive oxygen species or free radicals [8]. The short life of reactive oxygen species or free radicals constitutes a methodological obstacle, so it is feasible to study the activity of enzymes that form oxidants or neutralize them. Malondialdehyde (MDA) is the final product of lipid peroxidation and can be used as a marker of oxidation [9]. Numerous studies have evaluated MDA levels in diverse psychiatric disorders and especially in schizophrenia. Although results are inconsistent, a meta-analysis demonstrated increased MDA levels in schizophrenia [10].

Myeloperoxidase (MPO) is a crucial enzyme whose main function is production of hypochlorous acid to defend the body against pathogens. Its oxidant and inflammatory characteristics make MPO a potential marker of inflammation and oxidation. Much evidence denotes that schizophrenia has an inflammatory and oxidative basis, important in the disorder’s pathophysiology [11,12]. Several studies have identified changes in MPO levels in psychiatric disorders. MPO has been defined as a marker of immune activation for major depression [13], and higher MPO levels were reported in depressed bipolar patients [14]. Furthermore, increased MPO levels have been reported in schizophrenia [15]. Catalase (CAT) is an important antioxidant enzyme that catalyzes formation of water and oxygen from hydrogen peroxide. By reducing hydrogen peroxide anions, CAT prevents harmful oxidative effects [16]. In contrast to MPO, CAT has been widely investigated in schizophrenia, with decreased, unaltered, and increased levels reported [17,18,19].

Despite extensive investigation of enzyme activity in psychiatric disorders, little is known about their diagnostic value. A growing body of evidence indicates that redox imbalance and immune signaling are tightly interwoven in schizophrenia pathogenesis, whereby glutathione depletion leads to maladaptive inflammatory cascades [20]. In chronic medicated schizophrenia patients, plasma H_2_O_2_ levels were significantly elevated while total antioxidant capacity was reduced, correlating with symptom severity and suggesting ongoing oxidative stress despite treatment [21]. This is an emerging research area, with few studies addressing the diagnostic potential of enzyme activities. Bulut et al. highlighted high diagnostic activity of paraoxonase in generalized anxiety disorder [7]. Previous studies have mainly examined the diagnostic significance of these enzymes in bipolar disorder. Evidence regarding their role in schizophrenia remains limited. In fact, the diagnostic value of MPO in schizophrenia has been specifically investigated by Al-Asmari et al. [15], whereas the diagnostic utility of prolidase and CAT has been mainly studied in bipolar disorder [14,22]. Based on prior literature, this study aimed to investigate MPO and CAT levels and their diagnostic value in schizophrenia patients. While previous research has primarily explored the diagnostic utility of these enzymes in bipolar disorder, our study is the first to comprehensively assess their diagnostic value specifically in schizophrenia.

## 2. Methods

### 2.1. Study Population

Patients group consisted of individuals who were admitted to the Psychiatry Department of Dicle University Hospital and diagnosed with schizophrenia according to DSM-V criteria. Our study included 60 patients and 65 healthy control subjects. The control group consisted of healthy volunteers recruited from blood donors, none of whom had a personal or family history of psychiatric illness. Patients and controls were matched by age, gender, body mass index (BMI), and smoking habits. A comprehensive baseline characterization of the study cohort is provided in Table 1, including demographic and clinical variables such as age, sex, age at onset, illness duration, smoking status, body mass index (BMI), and major comorbidities. All patients included in the study were receiving antipsychotic treatment at the time of blood sampling; none were drug-naïve. The treatment regimens consisted of typical, atypical, or combined antipsychotic medications. Unfortunately, data on cumulative or lifetime antipsychotic dose (e.g., chlorpromazine equivalents), previous treatment switches, and concomitant medications were not available due to the retrospective design of the study. This limitation has been acknowledged in Section 4. All patients were clinically stable at the time of sampling and receiving antipsychotic treatment. The duration of illness and age at onset were recorded (Table 2), but the interval since the last psychotic episode was not systematically documented. All patients were on antipsychotic therapy, which may influence oxidative biomarker levels. Although cumulative dose data were unavailable, subgroup analyses by treatment type (typical, atypical, combined) consistently showed elevated biomarker levels compared to controls.

Patients with a history of drug abuse, chronic systemic diseases such as diabetes mellitus, hypertension, severe head injury or seizure disorders, vitamin B12 deficiency, schizoaffective disorder, or those using agents that may affect MPO, CAT, and MDA levels (e.g., beta-blockers such as carvedilol or nebivolol, statins, vasoactive medications, diuretics, and antioxidant drugs) were excluded from the study.

Additionally, among the 78 patients initially screened, 18 were excluded due to comorbidities—6 with diabetes mellitus, 5 with hypertension, and 7 with other chronic or systemic illnesses. Therefore, all included participants were free of metabolic disorders at baseline.

### 2.2. Clinical Assessment

Rating scales such as the Positive and Negative Syndrome Scale (PANSS) [23] and the Clinical Global Impression Severity of Illness Scale (CGI-S) [24] were applied to determine disorder severity. All patients were on antipsychotic medication. The Simpson-Angus (S-A) Scale was applied to evaluate pharmacotherapy-induced extrapyramidal symptoms (EPS) [25].

### 2.3. Blood Sampling and Processing

Twelve-hour fasting blood samples were taken from both the patient and control groups. Blood samples were collected in the morning between 8:00–9:00 a.m. after at least 12 h of fasting and non-smoking period, and drawn from the antecubital vein. Samples were transferred into heparinized blood tubes and centrifuged at 3000 rpm for five minutes in an ice medium within six hours at most, and sera were separated. Serum samples were stored at −80 °C until analysis. Blood samples were collected within the first 24 h after psychiatric admission, once patients were clinically stable and before any changes to their antipsychotic regimen.

MPO, CAT, and MDA levels were measured in serum samples in the Biochemistry Laboratory of Dicle University, Faculty of Medicine. MPO and CAT levels were determined using a sandwich ELISA method according to the manufacturer’s instructions. Briefly, serum samples were added to pre-coated wells, incubated with biotin-labeled antibodies, followed by streptavidin-HRP. After washing, chromogenic substrate was added and absorbance was measured at 450 nm. Commercial ELISA kits (Elabscience^®^, Houston, TX, USA) were used to measure serum MPO and CAT levels. Absorbance was measured using a BioTek ELx800 microplate reader (BioTek Instruments, Inc., Winooski, VT, USA).

MDA content was measured spectrophotometrically as described previously [1], using a Shimadzu UV-1201 spectrophotometer. The principle of the method was based on the spectrophotometric measurement of the color occurring during the reaction of thiobarbituric acid with MDA. Concentration of thiobarbituric acid reactive substances was calculated by the absorbance coefficient of the malondialdehyde–thiobarbituric acid complex and expressed in micromoles per liter. As a standard MDA, bis (dimethyl acethal)-TBA (thiobarbituric acid) complex was used. All reagents for the MDA assay were obtained from Sigma-Aldrich (St. Louis, MO, USA).

### 2.4. Ethical Approval

The diagnosis of schizophrenia was established by a psychiatrist according to DSM-V criteria. The study was designed prospectively and approved by the Clinical Research Ethics Committee of Dicle University Faculty of Medicine (27 November 2015/233). Written informed consent was obtained from all participants.

### 2.5. Statistical Analysis

All statistical analyses were performed using SPSS for Windows, version 24.0 (SPSS Inc., Chicago, IL, USA). Continuous variables were assessed for normality. Normality of data distribution was tested using the Shapiro–Wilk test. Parametric variables were compared between groups using the Student’s *t* test, while non-parametric variables were analyzed with the Mann–Whitney U test. Categorical variables were compared using the chi-square test. A two-tailed *p* value of <0.05 was considered statistically significant. A post hoc power analysis based on the observed effect size of MPO differences between patients and controls indicated a statistical power greater than 0.90 at α = 0.05.

Correlation analyses were conducted using Pearson correlation coefficients for parametric variables and Spearman correlation coefficients for non-parametric variables, with corrections applied for ties. Associations between each biomarker (MDA, MPO, CAT) and clinical symptom scores (PANSS, CGI) were evaluated using simple linear regression models, with biomarkers as independent variables and symptom scores as dependent variables. Receiver operating characteristic (ROC) curve analysis was performed to determine the optimal cut-off points for serum myeloperoxidase (MPO), catalase (CAT), and malondialdehyde (MDA) levels. The area under the curve (AUC) was calculated to assess diagnostic performance, including sensitivity and specificity. Positive and negative predictive values were also calculated. In addition, multi-ROC curve analysis was conducted to evaluate the combined diagnostic value of MPO, CAT, and MDA.

For multiple group comparisons, Bonferroni correction was applied to adjust for type I error. All statistical tests were two-tailed. Although multivariate adjustment for all confounding variables was not feasible, patients and controls were matched for age, sex, BMI, and smoking status, and subgroup analyses by medication type were performed. In this study, oxidative stress biomarkers (MDA, MPO, CAT) were treated as dependent variables, while diagnostic group (schizophrenia vs. control) and antipsychotic treatment type (typical, atypical, combined) served as independent variables. Comparisons were performed between schizophrenia patients and controls, as well as between each treatment subgroup (typical, atypical, combined) and controls for each biomarker. PANSS and CGI scores were considered dependent variables in correlation analyses, with biomarker levels as independent variables. Demographic variables such as age, sex, BMI, and smoking status were matched between groups and therefore not included as covariates.

The primary outcome of this study was the diagnostic performance of MPO, CAT, and MDA biomarkers individually and in combination. Secondary outcomes included the correlation of these markers with clinical severity measures (PANSS and CGI).

## 3. Results

While comparing patients and controls in terms of age, gender, smoking habits and body mass index we didn’t find any significant difference, see Table 1.

Detailed clinical specifications of schizophrenia patients are demonstrated in Table 2.

We found MDA levels were significantly higher in schizophrenia patients than healthy controls, means were 5.64 pg/mL and 3.42 pg/mL respectively, *p* < 0.001. We compared subgroups of patients taking atypical, typical and combined antipsychotics with healthy controls separately. All of the patient groups demonstrated increased MDA levels when compared to healthy controls. We compared MPO levels of patients and controls, medians were 77.25 ng/mL and 31.42 ng/mL respectively, *p* < 0.001. During the comparison of subgroups of patients with control group we used Mann Whitney-U test and Bonferroni Correction. Given the comparison among four groups (patients receiving typical, atypical, or combined antipsychotic treatment, and healthy controls), a *p*-value below 0.0125 was considered statistically significant. In this context, we found all of the patient groups demonstrated significantly higher MPO values than healthy controls. CAT activity was significantly higher in schizophrenia patients than healthy controls, medians were 22.06 ng/mL and 6.58 ng/mL respectively, *p* < 0.001. As for MPO, we accepted *p* value under 0.125 significant after Bonferroni Correction. We found CAT levels were significantly higher in every patient group compared to controls, *p* < 0.001 for all of them, see Table 3. These findings suggest that elevated oxidative stress markers are consistent across different antipsychotic treatment types, although the influence of cumulative dose could not be assessed.

**Figure 1:**
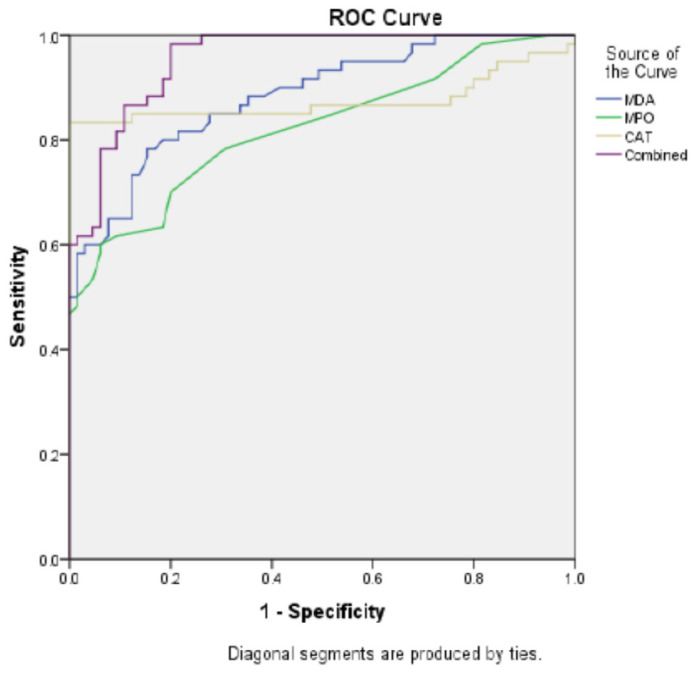
ROC Curve: Comparative Diagnostic Performance of IVDA, IVPO, CAT, and Combined Models.

To identify diagnostic value of MDA, MPO and CAT activity we performed ROC curve analysis. Area under curve (AUC) value was 0.882 and cut-off point was 34.56 ng/mL for MPO, *p* < 0.001. AUC value for CAT was 0.875 and cut-off point: 9.38 ng/mL, *p* < 0.001. AUC for MDA was 0.884 and the cut-off point was 3.79 pg/mL, *p* < 0.001. combined ROC curve of MDA, MPO and CAT showed better diagnostic performance, AUC: 0.955, cut-off point for combination was 43,943 ng/mL (Table 4 and Figure 1).

Among PANSS sub-scales, PANSS-N was correlated with only MPO, r value: 0.275 *p*: 0.033. CGI scores were correlated with both MPO and CAT, r values were 0.301 and 0.312, *p* values were 0.019 and 0.015 respectively. CAT showed a significant negative correlation with PANSS-T scores (r = −0.258, *p* = 0.046) (Table 5).

## 4. Discussion

Essential findings of our study are as following: increased MDA, MPO and CAT levels in overall schizophrenia patients and subgroups of patients. Additionally AUC values were 0.875 for CAT, 0.884 for MDA and 0.882 for MPO. Noteworthily combined ROC curve demonstrated better diagnostic performance, AUC was 0.995.

This study is an investigation that individually compares MPO levels in schizophrenia patients receiving typical, atypical, and combined antipsychotic treatments with those of healthy controls. Despite the critical role of MPO in oxidative stress, its levels have been investigated in psychiatric disorders only in a limited number of studies [15]. Consistent with our results, Al-Asmari et al. found MPO levels were increased in schizophrenia patients than healthy controls, 42.43 (U/L) and 30.37 (U/L) respectively [15]. Furthermore our study has some superiorities like larger sample size, detailed clinical data of patients and inclusion of diverse medication types. Noteworthily we found MPO levels were about 50% higher in patients taking typical antipsychotics than patients on atypical antipsychotic treatment. Possible explanations for these results are, typical antipsychotics may cause an increase in oxidative stress by increasing MPO levels, or increased MPO is a trait of schizophrenia and typical antipsychotics fail to decrease it. However, there is evidence that typical antipsychotics may increase oxidative stress or fail to decrease it. Behl et al. propounded haloperidol may induce cell death, which may be prevented by vitamin E [27]. Likewise in a study by Park et al. haloperidol failed to decrease oxidative stress while atypical antipsychotics were beneficial in these terms [28]. According to mentioned studies, we assert both of our explanations may be possible.

Catalase activity has been investigated widely in schizophrenia patients. A study by Herken et al. pointed out significantly high CAT levels in schizophrenia patients who were under antipsychotic treatments [17]. Another study denoted unchanged CAT levels in drug free schizophrenia patients [29]. Furthermore, as presented by Raffa et al. antipsychotic treatment increased CAT levels in schizophrenia patients [18]. On the other hand, Selek propounded that the imbalance in oxidative stress or antioxidant mechanisms may be due to chronicity of the psychiatric disorders or a compensatory response [14]. While evaluating our data with existing knowledge, the increased CAT activity may be due to antipsychotic effect or the chronicity of schizophrenia. MDA, a final product of lipid peroxidation, was significantly elevated in our patients, supporting the involvement of oxidative membrane damage in schizophrenia pathophysiology. This finding aligns with previous meta-analyses reporting increased MDA levels in schizophrenia.

The AUC values for CAT, MPO and MDA were 0.875, 0.882 and 0.884 respectively, all of them significant. This finding suggests that integrating multiple oxidative and inflammatory markers into a single diagnostic panel could significantly improve early detection and differentiation of schizophrenia from other psychiatric conditions. Such a multimarker approach may facilitate more accurate risk stratification and personalized treatment planning in clinical practice. The AUC value for combination of CAT, MPO and MDA was 0.995, which is higher than each of them. The AUC value between 0.8–0.9 predicts good diagnostic value but 0.6–0.7 represents poor diagnostic performance. Depending on this, we consider CAT, MPO and MDA activities show good diagnostic performance in patients who are under antipsychotic treatment. Attentively, combination of all parameters performed very good diagnostic performance. Sensitivity increased to 98.3%. Our study is the first to perform combined ROC curve to multiple oxidative-antioxidant parameters. The increased diagnostic value after combination of multiple parameters is important and further studies should focus on in diverse psychiatric disorders. As this topic keeps drawing attention, there are few studies evaluating the diagnostic performance of oxidants and/or antioxidant enzyme activities. Detailed information represented in Table 4 in this regard. In generalized anxiety disorder diagnostic performance of paraoxonase have been studied and the AUC was 0.980 [7]. Likewise, increased prolidase activity found to be highly diagnostic in schizophrenia and bipolar disorder [20]. Similar to our study, Selek et al. investigated CAT and MPO levels in bipolar patients and denoted an AUC value of 0.989 for CAT, 0.626 for MPO [14]. Taking into account that schizophrenia and bipolar disorder share common genetic or biochemical basis, high diagnostic values of CAT for both disorders may be meaningful. Because all patients were under antipsychotic treatment, the diagnostic performance observed may partly reflect medication effects. Future studies including drug-naïve individuals are necessary to differentiate disease-specific biomarker changes from treatment-related alterations.

We found significant correlation between PANSS-N scale and MPO rather than PANSS-P and PANSS-G. In addition, we found significant negative correlations between CAT and both PANSS-T and CGI scores. This suggests that enhanced antioxidant defense might be associated with lower overall symptom severity and better clinical status, highlighting a possible compensatory response in schizophrenia. We consider this finding is important for representing that increased oxidation-inflammation may have a potential for negative symptoms of schizophrenia. As far as we know, oxidative stress and inflammation may have crucial potential in schizophrenia pathogenesis specifically on negative symptoms [30,31]. Based on these evidence the correlation we found between PANSS-N scores (but not PANSS-P, PANSS-G or PANSS-T) and MPO are important. MPO takes part in neutrophils’ respiratory burst which is an indicator of inflammation and oxidation [32,33]. From this scope, increased MPO activity in schizophrenia patients and correlation between MPO and PANSS-N scores may be important in terms of suggesting a possible biochemical association between negative symptoms of schizophrenia and MPO.

In brief, we found MPO, CAT and MDA were increased in schizophrenia patients. The diagnostic value of combination of CAT, MPO and MDA was higher than their diagnostic value separately. Increased MPO could be important in terms of representing probable association between negative symptoms of schizophrenia and oxidation-inflammation. Our results should be considered as preliminary and need confirmation in larger samples and different laboratories. Given the limited number of female participants and the modest overall sample size, our findings should be interpreted as preliminary and hypothesis-generating rather than definitive.

### Limitations

This study has several limitations. The exclusion of drug-naïve schizophrenia patients makes it difficult to distinguish disease-specific alterations in oxidative biomarkers from those induced by antipsychotic treatment. Furthermore, the single-center design and relatively small sample size limit the statistical power and generalizability of the findings.

The measurement of biomarker levels at a single time point precludes the evaluation of dynamic changes related to disease progression or treatment response. Moreover, the absence of comprehensive metabolic, hematological, and immunological parameters restricts a deeper understanding of the oxidative and inflammatory mechanisms underlying schizophrenia. However, since all patients were under antipsychotic treatment, medication-related metabolic alterations, particularly the diabetes-inducing potential of some agents, might have contributed to oxidative biomarker changes.

Finally, the cross-sectional design does not allow causal inference; thus, the findings should be interpreted as associative rather than causal. Future multicenter prospective studies incorporating a broader range of clinical and laboratory indices are warranted to strengthen the mechanistic interpretation and external validity of these results.

## 5. Conclusions

In this study, we demonstrated that serum MDA, MPO, and CAT levels were significantly elevated in schizophrenia patients compared with healthy controls. The diagnostic accuracy of each parameter was good, but their combined evaluation yielded superior diagnostic performance, highlighting their potential utility as complementary biomarkers in schizophrenia. Importantly, MPO showed a significant correlation with negative symptom severity, suggesting a possible biochemical association between oxidative stress and the core psychopathology of schizophrenia. These findings support the role of oxidative imbalance and inflammation in the disorder’s pathophysiology and emphasize the importance of oxidative markers in diagnostic research. Larger, longitudinal, and multicenter studies including drug-naive patients are warranted to validate these results and further clarify the clinical applicability of oxidative stress biomarkers in schizophrenia.

## Figures and Tables

**Table 1:** Demographic and Clinical Characteristics of Patients and Controls.

	Patients (*n* = 60)	Controls (*n* = 65)	*p*
Gender M/F	46/14	51/13	0.651 ^a^
Smoker/Non smoker	37/23	46/19	0.282 ^a^
BMI (SD)	27.26 (5.5)	26.50 (3.1)	0.933 ^b^
Cigarettes p/d (SD)	20.92 (16.8)	20.58 (10.3)	0.752 ^b^
Age (SD)	33.67 (11.08)	33.71 (10.67)	0.943 ^b^

^a^ Chi-square test; ^b^ Independent samples *t*-test.

**Table 2:** Clinical Characteristics and Symptom Severity Scores of the Patient Group.

Age of Onset (SD)	Family History Yes/No	Duration of Illness (SD)	Number of Hospitalizations (SEM)	CGI (SD)	PANSS-P (SD)	PANSS-N (SD)	PANSS-G (SD)	PANSS-T (SD)
22.33 (7.94) ^b^	24/36 ^a^	12.58 (11.09) ^b^	1.98 (0.36) ^b^	3.58 (1.18) ^b^	21.22 (8.54) ^b^	21.63 (10.56) ^b^	44.87 (15.59) ^b^	88.70 (31.61) ^b^

SD: Standard Deviation, SEM: Standard Error of Mean, CGI: Clinical Global Impression Scale, PANSS-P: Positive and Negative Syndrome Scale–Positive, PANSS-N: Positive and Negative Syndrome Scale–Negative, PANSS-G: Positive and Negative Syndrome Scale–General Psychopathology, PANSS-T: Positive and Negative Syndrome Scale–Total Score. ^a^ Chi-square test; ^b^ Independent samples *t*-test.

**Table 3:** Oxidative Stress Biomarkers (MDA, MPO, CAT) in Patients Receiving Different Antipsychotic Treatments Compared to Controls.

	(1) Patients Overall (*n* = 60)	(2) Typical AP Treatment (*n* = 11)	(3) Atypical AP Treatment (*n* = 32)	(4) Combined AP Treatment (*n* = 17)	(5) Controls (*n* = 65)	Comparison	*p* Value-z Value
^a^ MDA pg/mL (mean ± SD)	5.64 ± 2.42	6.93 ± 3.22	5.05 ± 1.59	5.93 ± 2.88	3.42 ± 0.56	1-5 2-5 3-5 4-5	<0.001 −7.393 <0.001 −5.042 <0.001 −5.947 <0.001 −4.341
^b^ MPO ng/mL Median 25–75%	77.25 37.70–131.94	100.53 81.68–279.49	69.11 37.70–99.63	79.10 25.13–163.36	31.42 18.85–37.70	1-5 2-5 3-5 4-5	<0.001 −6.231 <0.001 −4.879 <0.001 −5.075 <0.002 −3.121
^b^ CAT ng/mL Median 25–75%	22.06 13.44–27.49	14.45 6.66–25.99	25.45 18.58–27.87	17.90 12.73–25.26	6.58 5.88–7.64	1-5 2-5 3-5 4-5	<0.001 −7.225 <0.001 −3.063 <0.001 −6.714 <0.001 −4.341

MDA: Malondialdehyde; MPO: Myeloperoxidase; CAT: Catalase. ^a^ Independent samples *t*-test, ^b^ Mann–Whitney U test, Significance was accepted at *p* < 0.05.

**Table 4:** Diagnostic performance of oxidative stress biomarkers in schizophrenia, bipolar disorder, and generalized anxiety disorder.

Disorder	Schizophrenia				Bipolar Disorder		Schizophrenia	Bipolar Disorder	GAD
Parameter	CAT	MDA	MPO	Combined	CAT	MPO	Prolidase	Prolidase	Paraoxonase
**Cut-off Point**	9.38 Ng /mL	3.79 pg/mL	34.56 ng/mL	43.943 ng/mL	14.12 IU/mg	n.r.	392.65 U/L	502.9375 U/L	7.740 μmol/L
**AUC**	0.875	0.884	0.882	0.995	0.989	0.625	1.0	0.989	0.980
**Sensitivity %**	83.3	80	78.3	98.3	n.r.	n.r.	100	n.r.	n.r.
**Specificity %**	100	81.5	69.2	80.0	n.r.	n.r.	100	n.r.	n.r.
**PPV %**	100	80	70.1	81.9	94.5	n.r.	100	98.5	92.5
**NPV %**	86.7	81.5	77.6	98.1	100	n.r.	100	92.4	90.0
***p* value**	<0.001	<0.001	<0.001	<0.001	<0.001	n.r.	<0.001	<0.001	<0.001
	**Our study**				**Selek et al. [14]**		**Gunes et al.** [26]	**Selek et al.** [22]	**Bulut et al. [7]**

AUC: Area Under Curve, PPV: Positive Predictive Value, NPV: Negative Predictive Value, GAD: Generalized Anxiety Disorder, MDA: Malondialdehyde, MPO: Myeloperoxidase, CAT: Catalase, r: Correlation Coefficient, n.r.: not relevant.

**Table 5:** Correlation of Oxidative Stress Biomarkers (MDA, MPO, CAT) with Clinical Symptom Scores.

	MDA	MPO	CAT
PANSS-T	n.s.	n.s.	r = −0.258 *p* = 0.046
PANSS-P	n.s.	n.s.	n.s.
PANSS-N	n.s.	r = 0.275 *p* = 0.033	n.s.
PANSS-G	n.s.	n.s.	n.s.
CGI	n.s.	r = 0.301 *p* = 0.019	r = −0.312 *p* = 0.015

n.s.: Not significant.

## Data Availability

The data supporting the findings of this study are available from the corresponding author upon reasonable request. The data are not publicly available because of privacy and ethical restrictions.
